# Engineering Antivenom: Research Progress and Future Directions in Snakebite Envenoming Therapy

**DOI:** 10.1111/nyas.70385

**Published:** 2026-08-26

**Authors:** Andreas H. Laustsen

**Affiliations:** ^1^ Department of Biotechnology and Biomedicine Technical University of Denmark Kongens Lyngby Denmark

**Keywords:** antibody discovery, antivenom, monoclonal antibodies, nanobodies, recombinant antivenom, snakebite envenoming, toxins, venom

## Abstract

Snakebite envenoming remains a major neglected tropical disease, causing substantial mortality and morbidity worldwide. While plasma‐derived antivenoms have been the mainstay of treatment for over a century, they present important limitations related to safety, efficacy, specificity, scalability, and accessibility. Recent advances in biotechnology, venom and toxin characterization, and antibody engineering have enabled the development of new types of envenoming therapeutics, including recombinant antivenoms based on monoclonal antibodies and nanobody‐based formats, as well as small‐molecule inhibitors. These approaches are supported by advances in toxicovenomics, structural biology, and diagnostic technologies, which together facilitate a more rational, toxin‐centric design paradigm. This review provides an overview of recent progress in the development of novel types of antivenom therapy, highlighting key design principles, emerging therapeutic modalities, and enabling technologies. Current challenges related to efficacy, breadth of coverage, manufacturability, and clinical translation are discussed, along with future perspectives for the development of safe, effective, and accessible treatments for snakebite envenoming.

## Graphical abstract

Snakebite envenoming therapy is evolving from plasma‐derived antivenoms toward rationally engineered therapeutics. Advances in toxicovenomics, structural biology, antibody engineering, computational protein design, recombinant expression, small‐molecule inhibitors, and diagnostics are enabling toxin‐centric development of safer, more effective, and accessible treatments. Integrating these complementary technologies provides a framework for new types of antivenoms with improved efficacy, broader coverage, scalable manufacturing, and enhanced clinical translation.

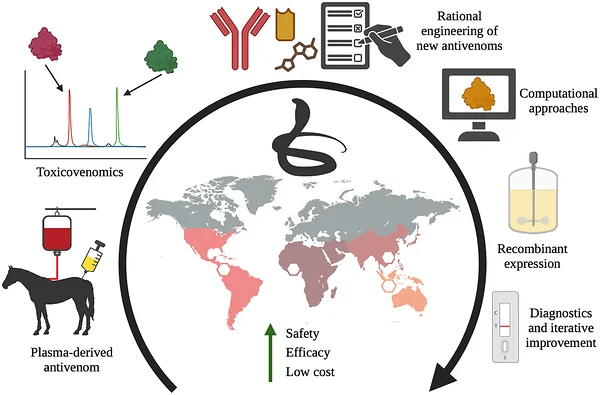

## 1 Introduction

**FIGURE 1:**
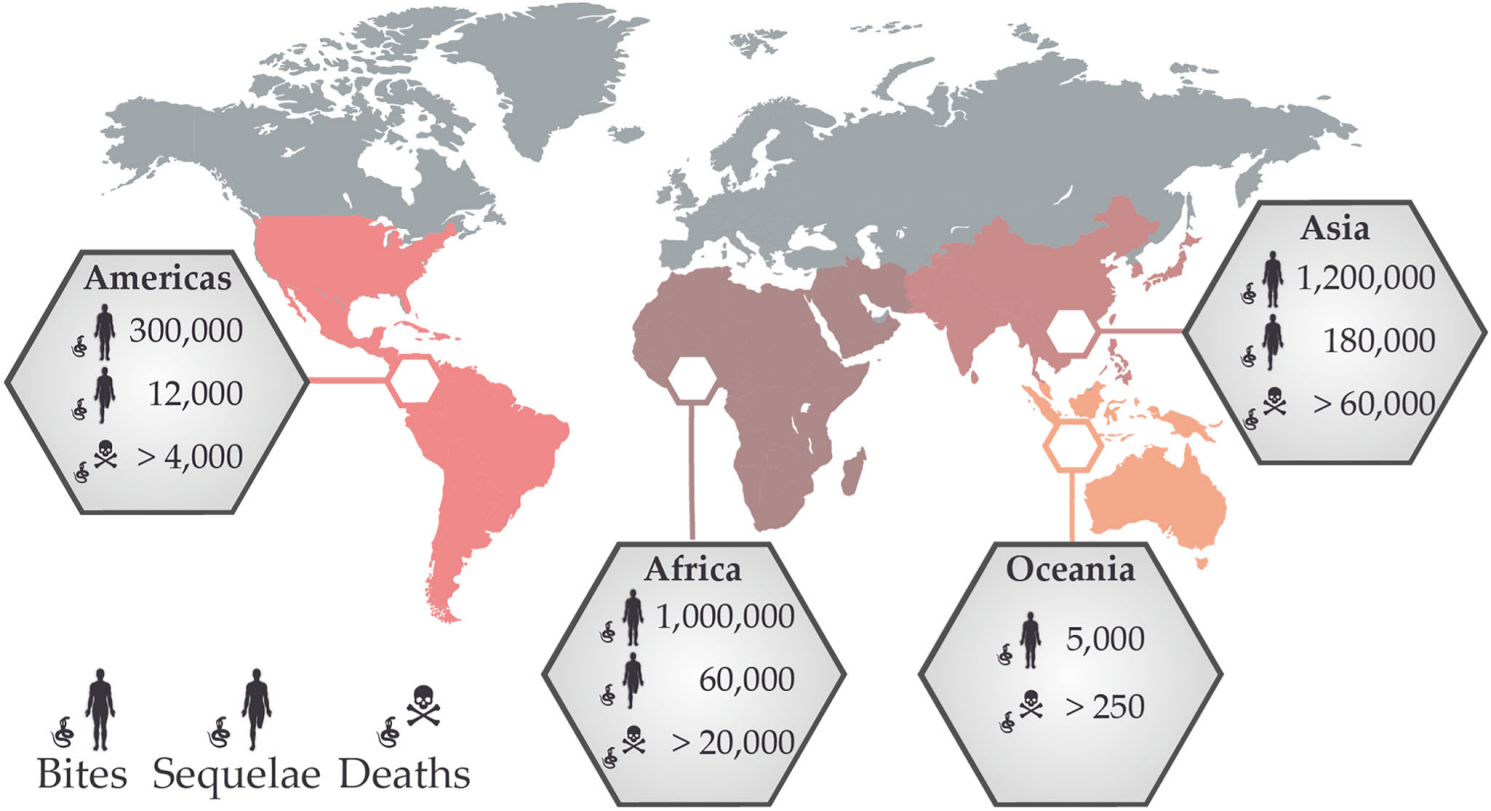
Schematic overview of the global snakebite burden, measured in number of bites, deaths, and sequelae per year. Numbers were obtained from Gutiérrez et al. [4]. Figure and legend recreated with permission from Laustsen [69].

Snakebite envenoming is a major, yet historically neglected, global health problem that continues to cause substantial mortality and morbidity, particularly in rural regions of sub‐Saharan Africa, South and Southeast Asia, and Latin America [1, 2, 3] (Figure 1). Each year, millions of snakebites are estimated to occur, resulting in hundreds of thousands of deaths and an even greater number of cases of permanent disability, including amputations, chronic kidney disease, and severe tissue damage [4]. In addition, many snakebite survivors experience long‐term psychological sequelae, including post‐traumatic stress disorder, depression, anxiety, and reduced quality of life, further increasing the individual and societal burden of snakebite envenoming [5, 6, 7]. The burden falls disproportionately on impoverished rural populations, many of whom rely on agricultural work and have limited access to timely medical care. Consequently, snakebite envenoming is not only a medical emergency, but also a major socioeconomic challenge that contributes to persistent poverty in affected communities [8, 9, 10, 11].

For more than a century, plasma‐derived antivenoms have remained the mainstay of snakebite treatment [12, 13]. These therapies are produced by immunizing animals, typically horses or sheep, with snake venoms and subsequently purifying polyclonal antibodies from their plasma [14, 15, 16, 17, 18]. When properly manufactured and administered, plasma‐derived antivenoms can effectively neutralize venom toxins and reduce mortality. However, despite their proven clinical utility, they suffer from important limitations. Because of their heterologous origin, plasma‐derived antivenoms are inherently immunogenic and may induce adverse reactions. Most are mild early hypersensitivity reactions or delayed serum sickness, whereas severe anaphylactic reactions are relatively uncommon with well‐manufactured modern antivenoms [19, 20, 21, 22]. In addition, they often contain a substantial proportion of non‐neutralizing antibodies, which thus necessitates high doses and increases treatment costs [23, 24, 25, 26]. Other challenges include batch‐to‐batch variability, dependence on reliable venom supply and animal immunization protocols, and manufacturing processes that are difficult to standardize and scale [17, 27, 28, 29, 30]. Furthermore, while many antivenoms are available as freeze‐dried formulations that reduce dependence on cold‐chain storage, liquid products still require refrigeration, and storage and distribution challenges continue to limit access in many low‐resource settings [17, 18]. Finally, although plasma‐derived antivenoms are generally effective at neutralizing circulating toxins and reducing mortality when administered promptly, they are often less effective at preventing or reversing local tissue damage, which can develop rapidly before adequate concentrations of antivenom reach the affected tissues [4, 30, 31]. This limitation contributes to the persistent burden of permanent disability following snakebite envenoming. Together, these limitations contribute substantially to the persistent burden of disability following snakebite envenoming and further highlight the need for safer, more effective, and more accessible therapeutic strategies.

Recent advances in biotechnology, antibody engineering, and venom characterization are beginning to reshape the field of antivenom research. In particular, toxicovenomics, recombinant protein production, and antibody discovery technologies such as phage display have enabled the rational development of new types of antivenom composed of defined toxin‐neutralizing molecules [15, 19, 28, 29, 32]. At the same time, emerging diagnostic approaches may improve the identification of envenoming, support patient stratification, and guide treatment decisions more effectively [33, 34, 35], which may influence which and how new antivenom products will be designed. This review examines these developments, with a particular focus on recombinant antivenoms, broadly neutralizing antibodies, novel antibody formats, and diagnostic tools that may improve the clinical management of snakebite envenoming.

## 2 Snake Venoms as Drug Targets

**FIGURE 2:**
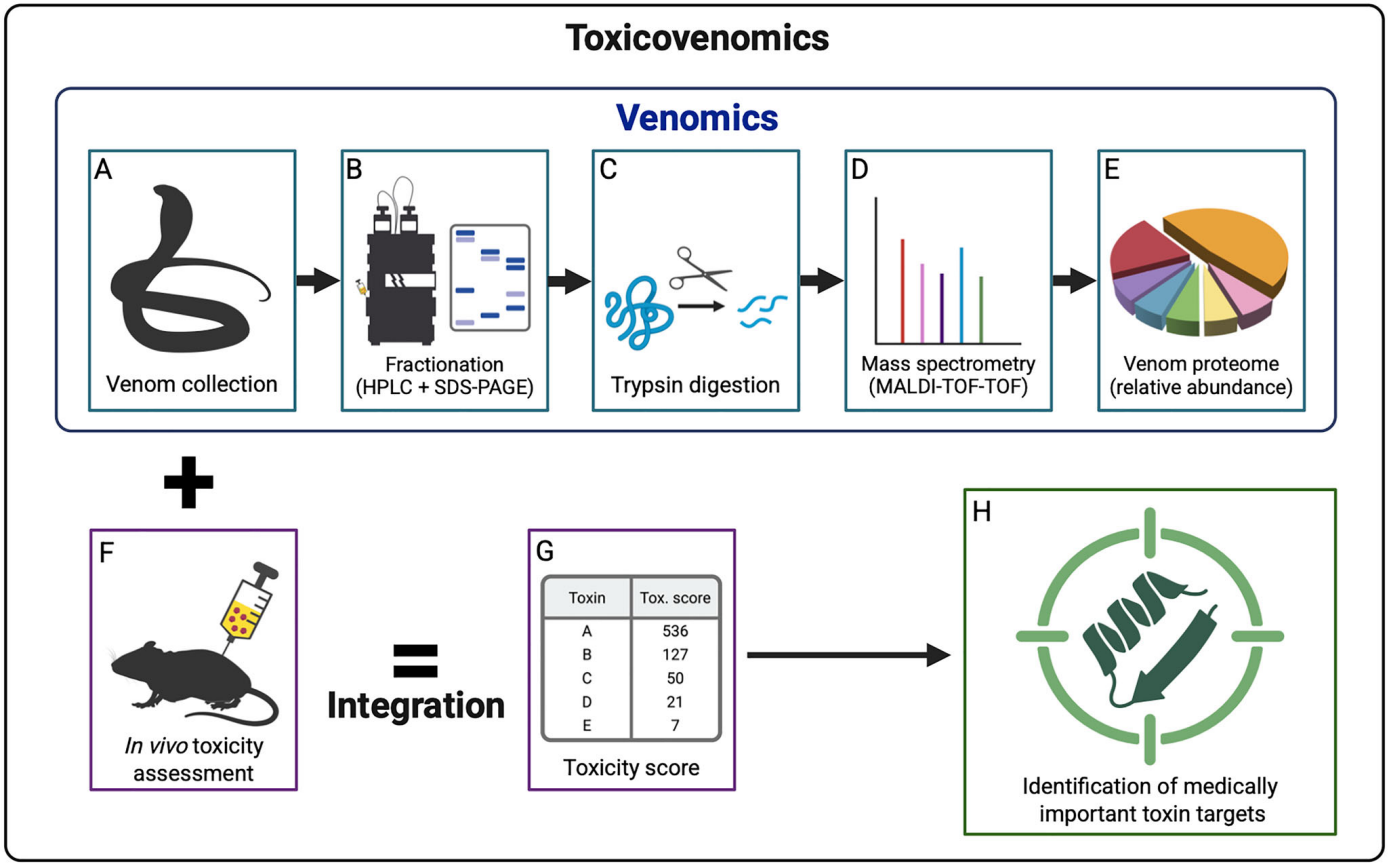
Overview of the venomics workflow and its extension, toxicovenomics. (A) Venom is collected from the snake. (B) The venom is separated into individual fractions using high‐performance liquid chromatography (HPLC) and sodium dodecyl sulfate‐polyacrylamide gel electrophoresis (SDS‐PAGE). (C) Proteins in each fraction are digested into smaller peptides using the enzyme trypsin. (D) The peptides are analyzed by mass spectrometry (e.g., MALDI‐TOF‐TOF) and identified using bioinformatic analysis. (E) The chromatographic and proteomic data are combined to determine the relative abundance of each toxin in the venom, generating a quantitative venom proteome. Toxicovenomics extends this workflow by adding (F) in vivo toxicity assessment of the venom fractions. (G) The toxicity data are integrated with the quantitative venomics data to assign a Toxicity Score to each toxin or toxin family. (H) This integrated analysis identifies the venom components that contribute most to clinical toxicity, thereby prioritizing the most important targets for antivenom development.

Venoms are complex mixtures of bioactive molecules that have evolved to disrupt key physiological processes in prey or predators [24, 36, 37]. From a therapeutic perspective, this complexity makes venoms particularly challenging drug targets. Individual venoms comprise diverse repertoires of toxins from multiple protein families, often acting through distinct molecular mechanisms and exhibiting substantial variation across species [4, 15, 38, 39]. Despite this diversity, only a subset of components is responsible for the most clinically relevant effects observed during envenoming [36, 40, 41, 42]. Identifying these medically important toxins is, therefore, a critical step in the development of new types of antivenoms, enabling a more focused and rational targeting strategy [41]. Advances in omics technologies, including venomics, antivenomics, and toxicovenomics, have enabled increasingly detailed characterization of venom composition and function [15, 43, 44, 45]. By integrating proteomic, transcriptomic, and functional data, these approaches facilitate identification of the toxins that drive toxicity (Figure 2). Importantly, toxin relevance is determined not only by intrinsic potency, but also by abundance within the venom. Quantitative frameworks such as the “toxicity score,” which integrate these parameters, allow toxins to be ranked according to their contribution to overall lethality and morbidity, thereby guiding target selection for therapeutic intervention [41, 46]. This has supported a shift from empirically derived antivenoms toward rationally designed therapies that prioritize neutralization of the most clinically significant toxin subsets.

In parallel, structural and molecular characterization of toxins has become increasingly important for guiding therapeutic design. High‐resolution structural data, combined with bioinformatic and biochemical analyses, enable identification of conserved epitopes across toxin families and species, which can be exploited for the development of broadly neutralizing antibodies or inhibitors [47, 48, 49, 50, 51, 52]. Building on this knowledge, recent studies using different discovery approaches have demonstrated that targeting structurally conserved regions of toxins can yield antibodies with broad cross‐reactivity and neutralizing capacity [48, 49, 53, 54, 55, 56, 57, 58]. This is particularly valuable given the high sequence variability among toxins, as structural conservation often provides a more reliable basis for achieving breadth of coverage. In addition, structural insights into toxin–antibody interactions can inform affinity maturation, optimization of binding modes, and possibly the design of multivalent or high‐avidity formats, thereby enhancing neutralization potency [47, 59].

**FIGURE 3:**
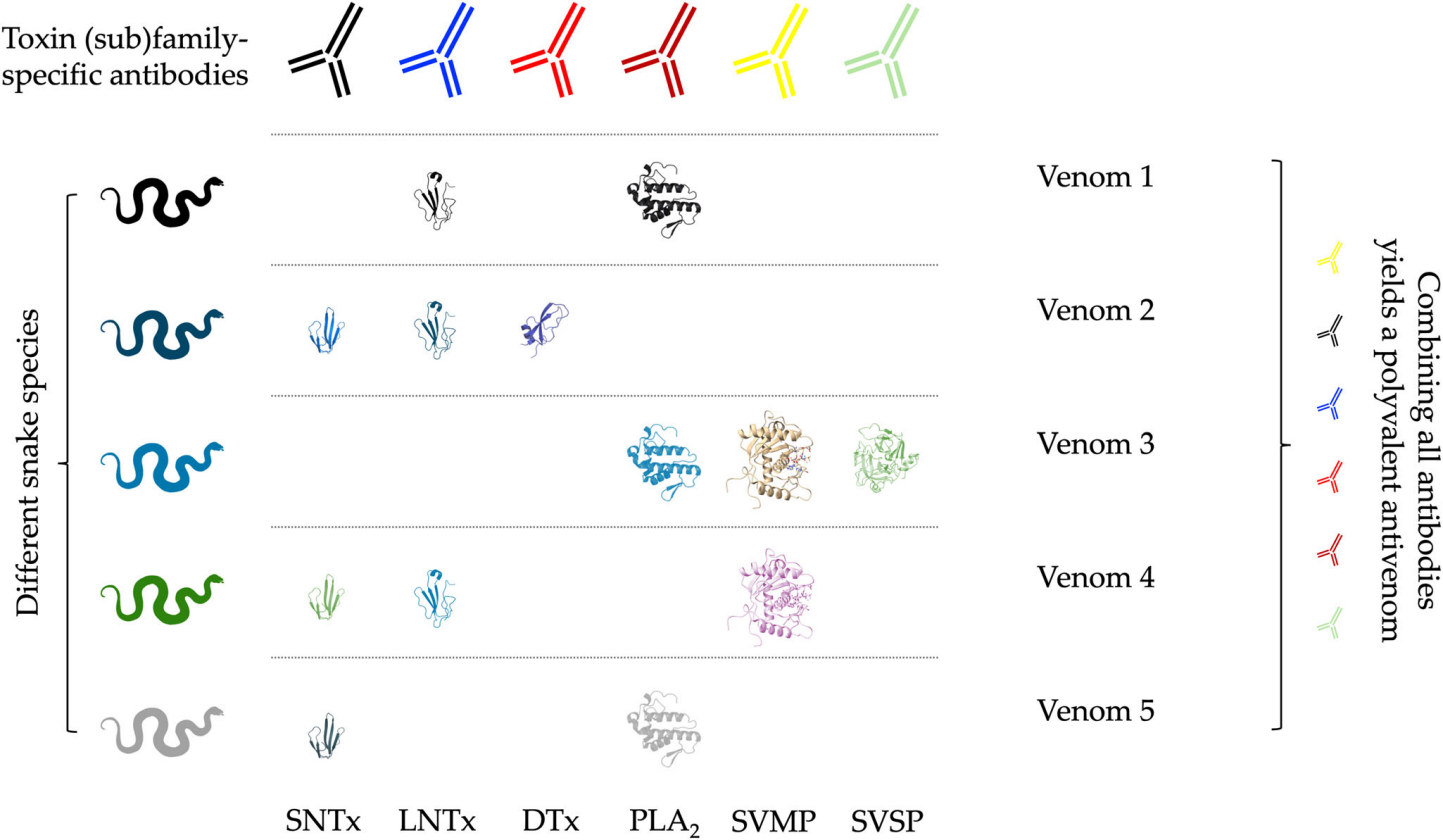
Schematic representation of how monoclonal IgG antibodies that are able to broadly neutralize entire toxin (sub)families can be used to design polyvalent recombinant antivenoms that are capable of cross‐neutralizing whole venoms from several different snake species. By combining the broadly neutralizing antibodies, it is possible to achieve broad species coverage with fewer antibodies. SNTx: short‐chain α‐neurotoxin, represented by short neurotoxin 1 (Uniprot ID: P01416) from *Dendroaspis polylepis*. LNTx: long‐chain α‐neurotoxin, represented by α‐elapitoxin‐Dpp2d (Uniprot ID: C0HJD7) from *D. polylepis*. DTx: dendrotoxin, represented by dendrotoxin 1 (Uniprot ID: P00979) from *D. polylepis*. PLA_2_: phospholipase A_2_, represented by myotoxin II (Uniprot ID: P24605) from *Bothrops asper*. SVMP: snake venom metalloprotease, represented by BaP1 (Uniprot ID: P83512) from *B. asper*. SVSP: snake venom serine protease, represented by jararacussin‐1 (Uniprot ID: Q2PQJ3) from *B. jararacussu*. Figure adapted with permission from Laustsen [69].

Although venoms are highly diverse, many clinically relevant effects can be attributed to a limited number of toxin families, including three‐finger toxins (3FTxs), phospholipases A_2_ (PLA_2_s), snake venom metalloproteases (SVMPs), and snake venom serine proteases (SVSPs), which underlie key pathological processes such as neurotoxicity, hematotoxicity, and tissue damage [4, 31, 51, 52, 60, 61]. The observation that a relatively small number of toxin classes account for most clinically significant effects supports the feasibility of targeted therapeutic strategies. Rather than neutralizing venoms indiscriminately, new approaches can be designed to selectively inhibit the principal drivers of toxicity, either individually or in combination [40, 53, 62, 63, 64]. This toxin‐centric and structure‐informed perspective has important implications for the design of novel envenoming therapies (Figure 3). It necessitates consideration of toxin diversity, redundancy, and synergistic interactions, as well as pharmacokinetic and pharmacodynamic constraints associated with different therapeutic modalities [24, 65, 66, 67, 68]. At the same time, achieving sufficient breadth of coverage across toxin variants and species while maintaining high specificity remains a key challenge. These considerations underpin the development of emerging therapeutic strategies, including small‐molecule inhibitors targeting conserved enzymatic functions and recombinant antibody‐based approaches providing highly specific neutralization. The following sections examine these modalities in detail, with emphasis on their design principles, advantages, and limitations.

## 3 Design Considerations Around Novel Envenoming Therapies

The development of new types of therapies for snakebite envenoming requires a transition from empirically derived treatment paradigms to a more rational, engineering‐driven design framework [69]. Within this framework, multiple parameters and aspects must be considered simultaneously, including efficacy, breadth of coverage, pharmacokinetics, manufacturability, shelf‐life, distribution, regulatory and clinical aspects, as well as business, financial, and possibly geopolitical factors and interests [69, 70, 71]. These parameters are inherently interdependent, and optimization of one may compromise another. Accordingly, the design of novel envenoming therapies can be viewed as a multiobjective optimization problem, where trade‐offs must be carefully balanced to achieve maximum clinical impact. Efficacy remains a central consideration and is typically assessed in preclinical models, where venom and antivenom are preincubated before the mixture is injected into a rodent. Unfortunately, these conventional assays for assessing efficacy may overestimate therapeutic performance, as they do not capture the dynamic interplay between toxin distribution and intervention in vivo [72, 73, 74]. Rescue‐type models, in which treatment is administered after a time delay following venom challenge, provide a more clinically relevant assessment by incorporating pharmacokinetic and toxicokinetic factors [73]. These include the rate of toxin release from the bite site, distribution of both toxins and antitoxins to target tissues, the onset of irreversible pathological effects prior to treatment, and the serum half‐lives of both toxins and antitoxins. Consequently, both the timing of administration and the ability of a therapeutic to intercept toxins in circulation or at tissue sites are critical determinants of efficacy.

Pharmacokinetic and pharmacodynamic properties further influence the suitability of different therapeutic formats [32]. Smaller molecules or antibody fragments may exhibit improved tissue penetration and distribution, which can be advantageous for neutralizing toxins acting locally at the bite site [27, 32, 55, 63]. In contrast, full‐length immunoglobulins with longer half‐lives may provide sustained systemic protection against circulating toxins [32]. The relationship between molecular format, distribution, and efficacy is, therefore, context‐dependent and must be evaluated in relation to the specific toxins targeted and their mechanisms of action [65, 72]. In addition, toxin synergism, where multiple toxins act in concert to enhance overall toxicity [66], further complicates therapeutic design and supports the need for multitarget intervention strategies.

Achieving sufficient breadth of coverage represents another key challenge for the design of new envenoming therapies [24, 47, 75, 76]. Given the substantial variation in venom composition not only between different snake species, but also between populations of the same species from different geographical regions, therapies often need to be tailored to the medically relevant snakes and venom profiles found in a particular region [24, 55]. This requirement underpins the development of broadly neutralizing agents and oligoclonal mixtures that aim to combine specificity with coverage [28, 40, 53, 55]. However, increasing breadth often necessitates inclusion of multiple components, which can increase manufacturing complexity and cost [77, 78]. Balancing minimal composition with adequate therapeutic coverage, therefore, remains a central design consideration [55, 79].

From a translational perspective, manufacturability and accessibility are critical. Snakebite envenoming predominantly affects populations in low‐resource settings, and new therapies must, therefore, be scalable and economically viable. Advances in recombinant protein production, antibody engineering, and small‐molecule development offer opportunities to improve consistency between batches and may reduce production costs and improve long‐term cost‐effectiveness, although these potential advantages remain to be demonstrated at commercial scale [19, 27, 32, 78]. At the same time, regulatory requirements, including demonstration of safety and efficacy and the establishment of appropriate preclinical and clinical evaluation frameworks, must be addressed to enable clinical translation.

Collectively, these considerations highlight the complexity of designing novel snakebite envenoming therapies. Successful strategies will require integration of toxin‐centric targeting with careful attention to pharmacological, technological, and socioeconomic constraints. The following sections examine emerging therapeutic modalities, including small‐molecule inhibitors and recombinant antibody‐based antivenoms, in the context of these design principles.

## 4 Small‐Molecule Inhibitors Against Snake Toxins

Small‐molecule inhibitors represent an emerging and complementary strategy for the treatment of snakebite envenoming, offering a mode of action distinct from antibody‐based therapies [27, 80, 81]. Rather than neutralizing toxins through molecular recognition in the manner of antibodies, the small‐molecule inhibitors reported to neutralize snake toxins typically inhibit enzymatic activity by binding to catalytic sites on toxins or by chelating essential metal ion cofactors. This makes them particularly well suited for targeting toxin families with conserved catalytic mechanisms, such as PLA_2_s, SVMPs, and (in theory) SVSPs, which play central roles in the pathology of many envenomings [82, 83].

A key advantage of small‐molecule inhibitors is their potential for broad‐spectrum activity. Because many enzymatic toxins share conserved active sites across venoms from different snake species, a single inhibitor may neutralize homologous toxins from multiple venoms. To an extent, this contrasts with antibody‐based approaches, where cross‐reactivity often requires explicit engineering [48, 49, 69]. Several compounds originally developed for other medical indications have demonstrated promise in this context, including PLA_2_ inhibitors, such as varespladib, and metalloprotease inhibitors, such as batimastat, marimastat, ethylenediaminetetraacetic acid (EDTA), and 2,3‐dimercapto‐1‐propanesulfonic acid (unithiol, DMPS), all of which inhibit key venom toxins in preclinical studies [19, 62, 63, 64, 84, 85]. Such drug repurposing strategies offer a relatively rapid pathway toward clinical translation, as aspects of their safety and pharmacokinetic profiles are already established [70].

Some small molecules may exhibit pharmacokinetic properties that are advantageous for neutralizing venom toxins, including good tissue distribution and, in some cases, oral bioavailability. These characteristics may facilitate early intervention, including potential use in prehospital settings where access to antivenom may be limited and personnel trained in antivenom administration unavailable [86]. Early administration may be critical for mitigating toxin‐induced pathology, especially for rapidly acting toxins or those that cause irreversible tissue damage [4, 64]. However, pharmacokinetic properties vary considerably between compounds, and some inhibitors, such as varespladib, have relatively short plasma half‐lives that may necessitate repeated administration to maintain therapeutic efficacy [20, 62, 87]. In this context, small‐molecule inhibitors may function as first‐line or adjunct therapies, delaying disease progression until definitive treatment can be provided [70].

Despite these advantages, important limitations must be considered. Effective small‐molecule inhibitors against snake toxins have generally only been reported against enzymatic toxins [19, 27, 81], reflecting both the current reliance on repurposed compounds with defined mechanisms and the intrinsic challenges of developing novel small molecules against structurally compact, nonenzymatic toxins such as three‐finger toxins, which offer limited opportunities for high‐affinity interactions and are major contributors to neurotoxicity in elapid envenomings. Consequently, small‐molecule inhibitors are unlikely to provide comprehensive therapeutic coverage across all medically relevant snake venoms in a given region if used alone. In addition, achieving sufficient potency and selectivity is inherently challenging, particularly when targeting structurally similar toxin variants while avoiding off‐target effects on endogenous human enzymes. Thus, although broad‐spectrum activity is advantageous, it may also reduce specificity, necessitating careful evaluation of safety profiles.

Integration into combination therapies represents an important strategy for maximizing the utility of small‐molecule inhibitors [50, 62, 63, 69, 88, 89]. Because venom toxicity is multifactorial, effective treatment often requires simultaneous targeting of multiple toxin classes. Small‐molecule inhibitors may, therefore, complement antibody‐based therapies by targeting conserved enzymatic toxins, whereas antibodies can provide broader coverage across both enzymatic and nonenzymatic venom components [24]. Such combination approaches may improve overall efficacy while reducing the required dose of individual agents.

In summary, the use of small‐molecule inhibitors in some cases represents a promising strategy for improving the treatment of snakebite envenoming, particularly as adjuncts to existing or emerging therapies. Notably, drug repurposing plays a central role in this space, offering a rapid and pragmatic pathway to clinical translation compared to the considerable challenges associated with de novo small‐molecule inhibitor development. Their potential for broad‐spectrum activity against enzymatic toxin families, favorable pharmacokinetics, and suitability for early intervention further support their inclusion in frameworks for the development of new snakebite interventions. However, limitations in toxin coverage and specificity underscore the need for careful consideration of when and how these agents should be integrated into multimodal treatment strategies.

Beyond small‐molecule inhibitors, other modalities, including synthetic peptides [90, 91], nucleic acid‐based aptamers [92], and antibody fragments delivered using mRNA and lipid nanoparticles [93], have also been explored for the neutralization of nonenzymatic toxins such as three‐finger toxins and noncatalytic PLA_2_s. However, these approaches remain at early stages of research and face significant development challenges, including short circulatory half‐lives, the need for pharmacokinetic optimization, and limited validation beyond proof‐of‐concept studies. In addition, it remains uncertain whether they can be manufactured at scale in a cost‐competitive manner. Consequently, although it is possible that some of these modalities could complement more established therapeutic approaches, their clinical utility in future snakebite envenoming therapy remains speculative [29].

## 5 Recombinant Antivenoms Based on Monoclonal Antibodies

**FIGURE 4:**
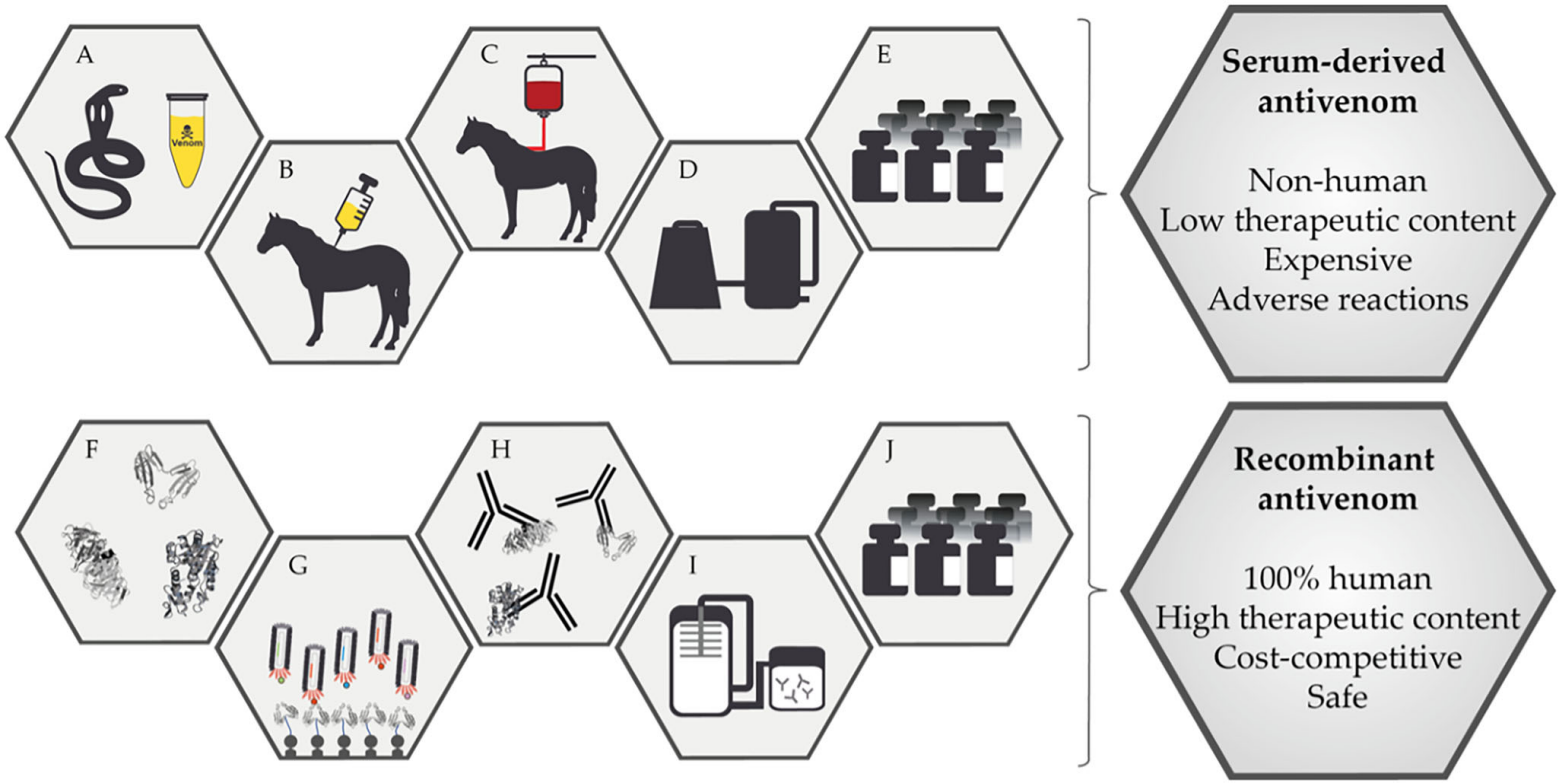
Schematic overview of the differences in the manufacturing processes between conventional serum‐derived antivenom and recombinant antivenom based on monoclonal antibodies. (A) Venom is obtained by milking a snake. (B) A production animal (typically a horse) is immunized using the snake venom. (C) After a lengthy immunization process, blood is drawn from the production animal. (D) The erythrocytes and plasma are separated, and IgG antibodies are isolated from the plasma using different precipitation techniques (in many cases, an enzymatic digestion step is also included to produce Fab or F(ab′)_2_ fragments). (E) After concentration and formulation, the antivenom is bottled and ready for distribution. (F) Medically important venom toxins are identified (e.g., by toxicovenomics). (G) Monoclonal antibodies are discovered against the medically relevant toxins (e.g., by phage display selection as shown). (H) Monoclonal antibodies are characterized, and an oligoclonal mixture of monoclonal antibodies is formulated and tested for neutralization capacity. (I) Using cell cultivation techniques, the oligoclonal antibody mixture is manufactured, for example, by using single‐batch expression technology. (J) After purification and formulation, the recombinant antivenom is bottled and ready for distribution. To the right in the figure, drawbacks of conventional plasma‐derived antivenoms are listed, together with the anticipated features of recombinant antivenoms based on human monoclonal antibodies that are expected to address these limitations. Many of these proposed advantages remain to be validated through clinical evaluation and industrial‐scale manufacturing. Figure and legend recreated with permission from Laustsen [69].

Recombinant antivenoms based on monoclonal antibodies or antibody fragments represent a promising alternative to conventional plasma‐derived products. In contrast to traditional antivenoms, which comprise heterogeneous mixtures of polyclonal antibodies, recombinant approaches enable the development of well‐defined oligoclonal mixtures targeting toxins of high medical relevance [94, 95] (Figure 4). This allows improved control over composition, specificity, and reproducibility, while reducing the risk of adverse reactions associated with heterologous animal proteins. A key advantage of monoclonal antibody‐based antivenoms is the possibility of engineering individual monoclonal antibodies with high affinity and specificity, as well as cross‐reactivity toward toxin variants [54, 56, 76, 96, 97]. By combining selected broadly neutralizing antibodies into rationally designed mixtures, particularly broad neutralization of complex venoms can be achieved while maintaining a limited number of components [53, 55, 98]. Recombinant production platforms further enable scalable and consistent manufacturing of such designed mixtures, with potential benefits for long‐term cost‐effectiveness. However, important challenges remain, including balancing breadth of venom and geographical coverage with mixture complexity and ensuring appropriate pharmacokinetic properties for effective toxin neutralization in vivo.

### 5.1 Discovery of Human Monoclonal Antibodies Against Snake Toxins

The discovery of human monoclonal immunoglobulin G (IgG) antibodies against snake toxins has been enabled by advances in antibody engineering and display technologies, particularly phage display [99, 100, 101, 102, 103]. These approaches allow in vitro selection of human monoclonal antibodies with defined binding properties from large and diverse libraries, thereby bypassing the need for animal immunization [104, 105, 106]. This represents an important step toward the development of fully human recombinant antivenoms with improved safety profiles.

A central challenge in antibody discovery for envenoming therapy is achieving broad neutralization across toxin variants and species [47, 50, 76, 107]. Even within the same toxin family, substantial sequence variability can limit antibody cross‐reactivity. To address this, strategies such as cross‐panning have been developed, in which antibody libraries are sequentially exposed to multiple related toxins [54, 76, 108]. This enriches for antibodies that recognize conserved structural features and increases the likelihood that these antibodies can cross‐neutralize multiple toxins from the same toxin (sub)family. Similarly, the use of consensus toxins, representing reconstructed or averaged sequences of toxin families, as antigens during an antibody discovery campaign can focus selection on conserved epitopes and facilitate the discovery of broadly neutralizing antibodies [109]. Finally, various affinity maturation strategies, such as light‐chain shuffling, are often required during antibody discovery to achieve sufficiently high affinity toward relevant toxin targets; importantly, while such antibody engineering efforts could in principle compromise breadth of neutralization, it has fortunately been shown that broad neutralization capacity is often preserved [47, 76, 110, 111, 112, 113].

Structural insights may also play an important role in guiding antibody discovery and optimization [47, 49, 50, 55]. High‐resolution characterization of toxin–antibody complexes can reveal the molecular basis of neutralization and identify critical interaction sites [110], which may further be used to improve affinity and specificity through engineering. Moreover, structural conservation across toxin families provides a basis for identifying epitopes suitable for targeting by broadly neutralizing antibodies [47, 50, 114, 115, 116]. Importantly, binding alone does not guarantee neutralization. Some antibodies may fail to inhibit toxin function or even enhance the toxicity of certain toxins [72]. Antibody discovery efforts must, therefore, incorporate functional screening and thorough preclinical validation to ensure that selected antibody candidates effectively neutralize their targets in experimental settings that as closely as possible reflect the pathophysiological context of snakebite envenoming. This typically involves in vitro assays assessing enzymatic inhibition, receptor binding blockade, or other relevant functional endpoints, as well as rescue‐type animal studies [73, 76, 107].

The development of oligoclonal antibody mixtures further emphasizes the need for rational antibody selection. By combining antibodies targeting different toxins or epitopes, multitarget neutralization of venom components can be achieved [23]. However, mixture composition must be carefully optimized to ensure sufficient coverage without unnecessary redundancy. In this context, the discovery of human monoclonal antibodies against snake toxins has played a key role in advancing recombinant antivenom development [117], with ongoing advances in antibody engineering, structural biology, high‐throughput screening, and computational methods expected to further improve the efficiency and effectiveness of these approaches.

### 5.2 Discovery of Single‐Domain Antibodies Against Snake Toxins

Single‐domain antibodies derived from camelid heavy‐chain‐only antibodies (such as V_H_Hs aka. nanobodies) have emerged as a promising alternative to conventional monoclonal antibodies for the neutralization of snake toxins [48, 49, 53, 55, 118, 119, 120]. Their small size (∼15 kDa), high stability, and ability to access cryptic epitopes make them particularly well suited for targeting venom components. Notably, single‐domain antibodies can bind conformational and recessed epitopes that are often inaccessible to larger antibody formats, which might be advantageous for neutralizing toxins with compact or structurally constrained active sites, including enzymatic toxins and receptor‐binding neurotoxins. In addition, their small size facilitates tissue penetration, potentially improving access to toxins acting at distal or poorly vascularized sites [55].

**FIGURE 5:**
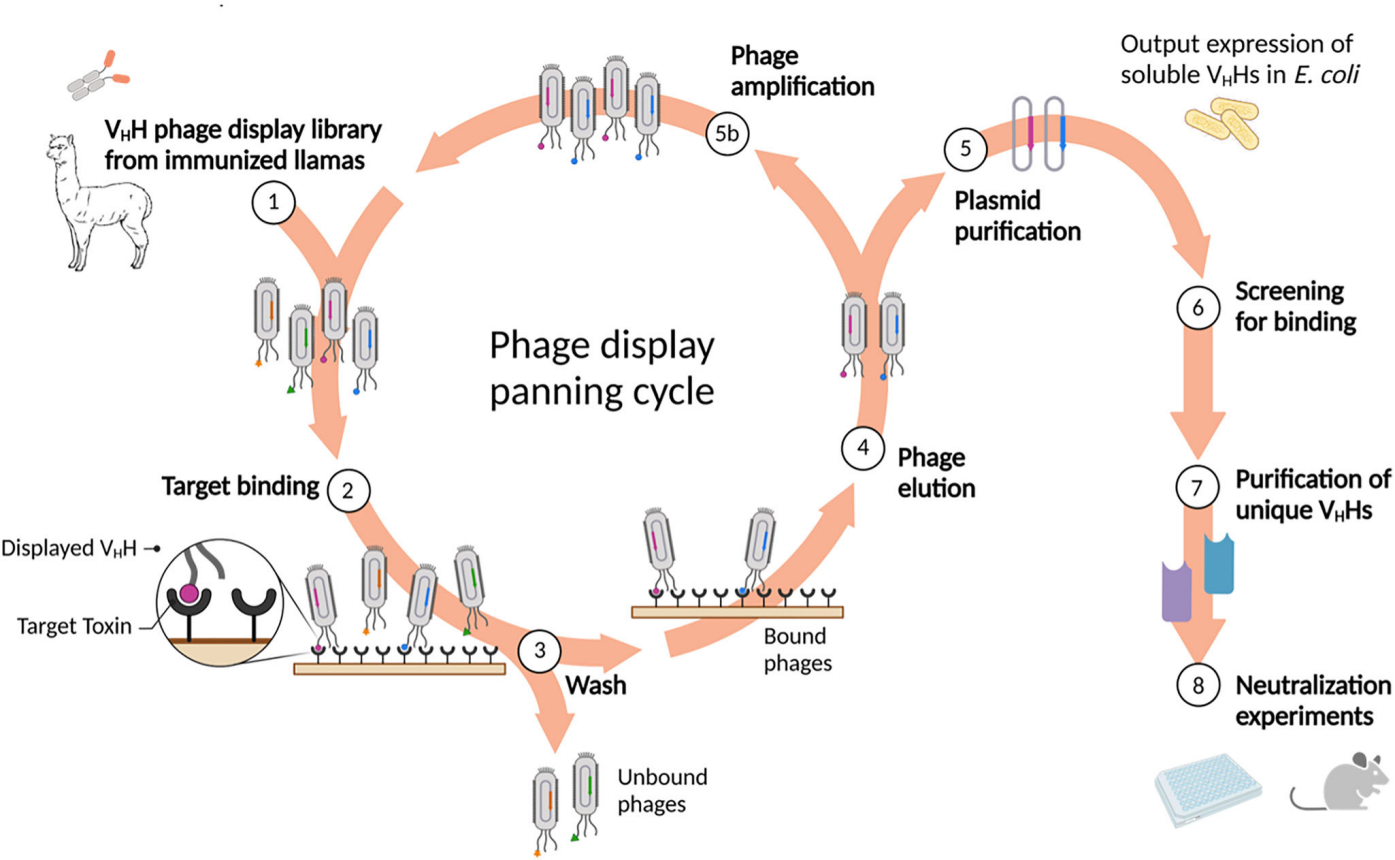
Schematic overview of the typical nanobody discovery pipeline. Figure is reprinted with permission from Melisa Benard Valle.

Single‐domain antibodies can be discovered from naive, immune, or synthetic antibody libraries [121]. Specifically, however, V_H_H discovery typically involves immunization of camelids followed by construction of V_H_H libraries and selection using display technologies [122, 123] (Figure 5). As with conventional antibodies, strategies such as cross‐panning and the use of consensus toxins as antigens can be applied to enrich for broadly neutralizing candidates [48, 49, 53, 55]. Another key advantage of single‐domain antibodies lies in their suitability for being engineered into multivalent or multispecific formats. By fusing with Fc domains or linking multiple single‐domain antibody domains, constructs can be generated with increased half‐life, avidity, or the ability to simultaneously target multiple toxins from entirely unrelated toxin (sub)families [53, 59, 118]. Such designs can be used to enhance neutralization potency and broaden toxin coverage, which is particularly relevant given the compositional complexity of venoms.

Despite these advantages, several challenges remain. The small size of single‐domain antibodies typically leads to rapid renal clearance and thus short half‐lives, which might limit the duration of their therapeutic effect to only a few hours. This can be problematic, as venom toxins may persist in the body due to sustained release from the bite site (venom depot effects) [32, 53, 124]. Strategies such as fusion to Fc domains or incorporation of albumin‐binding motifs can be used to extend their circulation time, although these modifications may alter other properties, including tissue distribution [53]. In addition, broadly neutralizing single‐domain antibodies can effectively target entire toxin (sub) families and may be deployed as oligoclonal mixtures, incorporated into conventional antivenoms, or used alongside other therapeutic modalities [24, 55]. Their modularity and ease of engineering further facilitate the design of combination strategies when broader coverage or complementary mechanisms of action are desired.

Overall, single‐domain antibodies represent a versatile antibody format for the development of new types of recombinant antivenoms. Their unique structural and functional properties complement those of conventional antibodies (immunoglobulin G) and provide new opportunities for developing recombinant antivenoms that might be possible to manufacture at lower cost.

### 5.3 Manufacturing Considerations

The successful translation of new types of therapies into clinical use depends not only on their therapeutic utility, but also on whether it is feasible to manufacture them scalably, robustly, and cost‐effectively [125, 126, 127]. This is particularly important in the context of snakebite envenoming, which predominantly affects populations in low‐ and middle‐income countries, where affordability and accessibility are critical determinants of clinical impact [69, 128]. Recombinant antivenoms based on oligoclonal mixtures of (human) monoclonal antibodies or antibody fragments (e.g., single‐domain antibodies) present both opportunities and challenges from a manufacturing perspective [78, 129]. A key advantage is the ability to produce well‐defined therapeutic mixtures with consistent composition and quality, thereby overcoming the batch‐to‐batch variability associated with plasma‐derived antivenoms [55, 77, 94]. However, conventional production strategies, where individual monoclonal antibodies are expressed, purified, and subsequently combined, are associated with substantial cost and process complexity, as each component requires separate upstream and downstream processing [77, 78].

To address this, alternative strategies based on cocultivation of multiple antibody‐producing cell lines have been developed [77, 130]. In these approaches, distinct cell lines expressing different antibodies or antibody fragments are combined within a single bioprocess, enabling simultaneous production of oligoclonal mixtures. Key to this concept is maintaining sufficient control over the relative abundance and productivity of each cell line to ensure consistent antibody composition, batch‐to‐batch reproducibility, and the desired neutralization profile of the final oligoclonal product. Experimental studies have demonstrated that stable cell line ratios and predictable antibody compositions can be maintained during cultivation, supporting the feasibility of scalable and reproducible manufacturing [77].

Beyond mammalian systems, microbial expression hosts offer additional opportunities for cost reduction [131]. In particular, one‐pot production of experimental oligoclonal nanobody‐based antivenoms has been demonstrated using engineered *Escherichia coli*, in which multiple V_H_H‐expressing strains are cocultivated to produce defined mixtures [130]. These systems can achieve high expression titers and enable simplified downstream processing, including extracellular secretion and streamlined purification. Technoeconomic analyses indicate that such approaches might be associated with substantially reduced manufacturing costs compared with conventional manufacturing approaches for IgGs, although these estimates remain to be validated at commercial scale [130].

Despite the abovementioned advances, several challenges remain. Ensuring consistent product composition across scales, maintaining the stability of oligoclonal mixtures, and meeting regulatory requirements for complex biologics are all nontrivial. In addition, differences between expression systems, such as glycosylation in mammalian cells versus its absence in some microbial systems, could influence pharmacokinetics and immunogenicity for some antibody formats, and must, therefore, be carefully evaluated [27]. Finally, manufacturing strategies and the format of the antivenom product must be aligned with intended use scenarios. This includes considerations of formulation stability, storage conditions, and distribution logistics, particularly in regions with limited cold‐chain infrastructure. Integration of process engineering, cost modeling, and product design will, therefore, be essential to ensure that new types of antivenoms are not only scientifically advanced, but also practically deployable at scale.

### 5.4 Preclinical Testing

Preclinical evaluation of novel envenoming therapies presents distinct challenges, particularly in translating findings from experimental models to human clinical settings. Murine models have traditionally served as the foundation of preclinical testing, providing important insights into venom toxicity and therapeutic efficacy [73, 74, 132]. However, their limitations are increasingly evident, especially in the context of new types of envenoming therapeutics such as recombinant antibodies and small‐molecule inhibitors against snake toxins [72, 124]. As discussed earlier, conventional murine preincubation assays may overestimate therapeutic efficacy because they fail to capture the pharmacokinetic and toxicokinetic complexity of clinical envenoming. While rescue‐type models improve translational relevance, murine systems still provide only limited insight into clinically relevant in vivo behavior and pharmacological performance [72, 73, 74]. Specifically, due to their small size, mice do not readily allow for serial blood sampling or detailed analysis of absorption, distribution, metabolism, and excretion processes. In addition, physiological differences between rodents and humans can limit the predictive value of these models for dosing and therapeutic efficacy. Consequently, reliance on murine lethality assays alone may provide an incomplete assessment of therapeutic performance [124, 132]. To address these limitations, there is increasing interest in large animal models, including sheep and pigs, which offer improved translational relevance [124]. These models enable more detailed pharmacokinetic analyses, such as evaluation of toxin absorption pathways (e.g., lymphatic vs. vascular uptake), as well as assessment of therapeutic distribution and clearance [124]. They also allow investigation of clinically relevant endpoints beyond survival, including tissue damage, coagulopathy, and recovery of physiological function. Integration of multiple preclinical models is, therefore, likely required to best capture the complexity of envenoming and its treatment. While murine models remain valuable for initial screening and proof‐of‐concept studies, large animal models provide a critical bridge toward clinical translation. Establishing standardized protocols and endpoints across models will be essential to improve comparability and reproducibility.

Overall, the development of preclinical testing strategies for new types of envenoming therapies should move toward more translationally relevant and mechanism‐informed evaluation frameworks, which are essential to support the development of safe and effective treatments.

## 6 The Role of Generative Protein Design in Antivenom Research

Recent advances in generative protein design, particularly those enabled by deep learning, have introduced new opportunities for the development of snakebite therapeutics [19, 133]. These approaches enable the de novo design of binding proteins that interact with specific toxin targets, providing an alternative to traditional discovery pipelines based on immunization or selection and screening of large antibody libraries [121]. In the context of snakebite envenoming, this might be particularly relevant for toxin classes that are poorly immunogenic and/or difficult to target using conventional in vitro discovery strategies [134].

A key strength of generative protein design is its ability to leverage structural and sequence information to directly engineer binding interfaces [135]. Computational frameworks such as RFdiffusion, RFdiffusion3, ProteinMPNN, LigandMPNN, and BindCraft allow the design of small, stable proteins that bind predefined regions of a toxin, including functionally critical epitopes involved in receptor interaction or membrane disruption and glycosylated sites [135, 136, 137, 138, 139]. Recent studies have demonstrated how binders targeting both short‐ and long‐chain α‐neurotoxins, as well as cytotoxins within the 3FTx family, can be designed, achieving high binding affinities and strong agreement between predicted and experimentally determined structures [133]. These binders can be designed to inhibit toxin function through steric mechanisms, for example, by blocking interactions with nicotinic acetylcholine receptors. In addition, emerging in silico methodologies for the design and optimization of single‐domain antibodies are beginning to complement these approaches and may be particularly relevant in this context [140, 141].

An additional advantage of computational approaches is the reduced reliance on extensive experimental screening. Conventional antibody discovery typically involves large libraries and iterative selection processes, which are resource‐intensive and dependent on toxin availability for the screening process [121, 133, 142]. In contrast, computational design enables the generation of candidate binders in silico, followed by targeted experimental validation, thereby accelerating early‐stage discovery and reducing associated costs. The resulting binding proteins are often small and highly stable, with properties such as thermal robustness, improved tissue penetration, and compatibility with microbial expression systems that may support cost‐effective manufacturing.

Despite these advantages, generative protein design should be considered complementary to established discovery methodologies, as some limitations remain. Successful design may depend on the availability of high‐quality structural or sequence data, which may be lacking for some medically relevant toxins. Moreover, high‐affinity binding achieved in silico or in vitro does not necessarily translate into in vivo efficacy, particularly in the context of complex venom‐induced pathologies, toxin synergism, and potential antibody‐mediated enhancement of toxicity [72, 73].

The high specificity of designed binders, while advantageous for precise targeting, may inherently constrain breadth of coverage across diverse toxin variants, thereby introducing a complex and computationally demanding requirement for extensive cross‐reactivity screening and optimization across diverse toxin variants to achieve sufficient neutralization of complex venoms. As with other therapeutic modalities, key parameters such as pharmacokinetics, immunogenicity, and delivery require careful evaluation [143]. Although the relatively small size of designed proteins may facilitate improved tissue penetration, it may also lead to rapid systemic clearance, potentially limiting their therapeutic window and necessitating half‐life extension strategies [32]. Furthermore, as many of these engineered proteins diverge from traditional biologics, regulatory frameworks governing their evaluation and approval remain in development, which may influence their translational trajectory. For this reason, many current research efforts within the computational sciences focus on in silico methods that can be used to design known and proven antibody formats, such as single‐domain antibodies [141].

Taken together, generative protein design constitutes a valuable and increasingly versatile addition to the recombinant antivenom development toolbox. Its capacity to generate binders against structurally and functionally challenging toxin classes provides distinct opportunities, particularly when used in conjunction with established approaches for antibody discovery. Combining generative protein design methods with protein structure prediction tools also enables the design of binders against very specific epitopes, as well as toxins for which only their DNA/RNA sequence exists (i.e., toxins that are difficult to isolate from venom, complicated to express recombinantly, or encoded by dormant genes in a snake genome) [133, 144]. In addition, in silico tools can be integrated with existing discovery pipelines to support the optimization and engineering of antibodies, for example, by guiding affinity maturation, improving stability, or enhancing cross‐reactivity. Rather than displacing established methodologies, in silico design is, therefore, likely to complement and inform these efforts, enabling the targeting of previously intractable toxins and facilitating the rational design of recombinant antivenoms, including combination strategies that may incorporate antibodies, antibody fragments, and small‐molecule inhibitors.

## 7 Snakebite Diagnostics in Relation to Antivenom Development

Accurate and timely diagnosis of snakebite envenoming is a critical component in the implementation of new types of envenoming therapies, including recombinant antivenoms and small‐molecule inhibitors. As treatment strategies evolve from predominantly mono‐ or polyvalent plasma‐derived products toward mechanism‐based and toxin‐targeted interventions, the importance of precise diagnostic tools is likely to increase. Diagnostics are, therefore, not only essential for clinical decision‐making, but also play a key role in enabling the rational development and effective deployment of these emerging therapies.

New types of envenoming therapies are typically designed to target defined toxin families or specific molecular epitopes. While this improves specificity and safety, it also creates a dependency on understanding which toxins are present and clinically relevant in a given envenoming [40, 41, 47, 49, 50, 55]. In contrast to plasma‐derived antivenoms, where broad polyspecificity may sometimes partially compensate for diagnostic uncertainty, more targeted therapies place greater emphasis on alignment between venom composition and therapeutic coverage. Accordingly, diagnostics capable of identifying the biting species/genus or, ideally, the dominant toxin classes involved may become increasingly important for optimizing treatment selection.

Recent advances in diagnostic technologies provide a foundation for this transition [33]. Immunoassay‐based platforms and emerging point‐of‐care tests have demonstrated the ability to detect venom antigens in biological samples, enabling rapid and potentially field‐deployable diagnostics [34, 35]. Building on these developments, there is growing interest in toxin‐resolved diagnostics capable of distinguishing between major toxin families, which may further support clinical decision‐making.

Beyond clinical decision‐making, diagnostics may also support the development and optimization of new types of envenoming therapies by enabling quantitative assessment of circulating toxins and thereby informing pharmacokinetic and pharmacodynamic analyses. This is particularly relevant for complex modalities such as antibody mixtures, where relationships between binding, toxin abundance, and in vivo efficacy remain to be defined beyond the preclinical setting, as well as for small‐molecule inhibitors, where temporal aspects of inhibition may be critical. However, important challenges remain, including the need for sensitive, specific, and robust tools suitable for low‐resource settings, as well as the considerable diversity of venom compositions across species and regions. Additional challenges relate to the validation and translation of such diagnostic tools. Access to well‐characterized clinical samples remains limited, and the lack of reliable reference standards or confirmatory methods complicates benchmarking of assay performance in both laboratory and field settings. Furthermore, the absence of standardized guidelines or target product profiles for snakebite diagnostics leaves key performance criteria, such as acceptable sensitivity, specificity, and limits of detection, insufficiently defined, requiring developers to navigate these aspects with limited guidance. Beyond the scientific challenges, regulatory pathways are complex and geographically variable, meaning that successful implementation will depend not only on robust validation but also on navigating diverse approval frameworks before these diagnostics can reach clinical use. Overall, while diagnostics have traditionally played a more supportive role, their importance is likely to increase alongside the development of more targeted therapeutic approaches, both in enabling rational design of envenoming therapies and in supporting clinical implementation. However, whether these diagnostic tools will be widely adopted will depend not only on their analytical and clinical performance but also on demonstrating cost‐effectiveness relative to existing syndromic approaches, particularly in low‐resource settings.

## 8 Current Challenges and Future Outlook

Despite significant advances in the development of new types of therapies for snakebite envenoming, substantial challenges remain before these approaches can achieve widespread clinical implementation [19, 69, 117]. These challenges span scientific, technological, regulatory, clinical, socioeconomic, and business domains, reflecting the complexity of translating innovative concepts into accessible treatments for a neglected tropical disease.

A central scientific challenge still lies in the diversity and variability of venoms across species and geographical regions [24]. Although toxin‐centric approaches have shown that a limited number of toxin families account for most clinically relevant effects, achieving sufficient breadth of coverage remains challenging [47, 50, 53, 55]. Recombinant antivenom products based on monoclonal antibodies or antibody fragments must balance neutralization capacity and specificity with cross‐reactivity, often requiring carefully optimized oligoclonal mixtures. Similarly, small‐molecule inhibitors targeting conserved enzymatic functions may not provide comprehensive coverage against nonenzymatic toxins. These limitations highlight the need for careful design of antivenom products that are optimized for the range of medically relevant snake species within a given region, acknowledging that broadly acting therapies will inevitably represent a compromise and may not be perfectly suited for any individual envenoming.

Another key challenge concerns translation from preclinical models to clinical efficacy [145]. While many new types of experimental envenoming therapies have demonstrated promising results in vitro and in rodent models, their performance in human envenoming cases remains uncertain. Differences in toxicokinetics, pathogenesis, and patient variability complicate extrapolation from experimental systems. Improved preclinical models and integration of pharmacokinetic and pharmacodynamic data will, therefore, be essential to guide clinical development [124].

From a technological perspective, manufacturing and cost remain critical considerations [78, 79, 129, 146, 147]. Although recombinant production platforms offer advantages in consistency and scalability, development costs and infrastructure requirements can be substantial. Innovations such as single‐batch expression systems and microbial production platforms may reduce costs, but further optimization and scale‐up are needed [77, 130]. Ensuring affordability and accessibility in low‐resource settings will be a key determinant of clinical impact.

Regulatory pathways for novel therapeutic formats, including recombinant antibody mixtures, nanobodies, and de novo designed binding proteins, also present challenges. These types of products may not align with existing frameworks for plasma‐derived antivenoms nor traditional biologics, likely necessitating adapted regulatory guidelines. In addition, demonstrating safety and efficacy for complex, multicomponent therapies will require carefully designed clinical trials and standardized evaluation criteria.

Integration of diagnostics and therapeutics represents another important area of development. As treatments could be designed to be more targeted, accurate identification of the perpetrating snake species in an envenoming case becomes increasingly important. However, reliable, field‐deployable diagnostic tools remain limited [33]. Addressing this gap will be essential for enabling optimal treatment strategies and maximizing therapeutic effectiveness.

Looking forward, the field has an opportunity to move toward a more modular and rational design paradigm, in which therapies are assembled based on defined toxin targets and tailored to regional needs. Advances in antibody engineering, small‐molecule discovery, and computational protein design continue to expand the available toolkit, enabling the development of interventions that are safer, more potent, and have broader species coverage. In parallel, improvements in manufacturing technologies and cost modeling are bringing these approaches closer to practical implementation.

Ultimately, successful translation will depend on coordinated efforts across academia, industry, regulatory agencies, global health organizations, (semi‐)philanthropic funding bodies, and investors. While conventional antivenoms are likely to remain part of the therapeutic landscape in the near term, new approaches have the potential to substantially improve safety, efficacy, and accessibility. Continued investment in research, product development, scale‐up, and implementation strategies will be essential to realize this potential and reduce the global burden of snakebite envenoming.

## 9 Conclusion

Snakebite envenoming remains a major global health challenge, but recent advances in biotechnology are reshaping how this condition can be understood and treated. A transition is underway from empirically derived, animal‐based therapies toward rationally designed, mechanism‐based interventions, driven by improved insight into venom composition and function. Central to this shift is the recognition that a limited number of toxin families account for most of the clinically relevant effects of snake venoms, enabling more focused and efficient therapeutic strategies [19, 24, 40, 50, 55]. Recombinant antivenoms based on monoclonal antibodies or antibody fragments represent a key component of this emerging paradigm, offering defined composition, improved safety, and the potential for scalable manufacturing. In parallel, small‐molecule inhibitors provide complementary approaches for targeting conserved enzymatic toxins, particularly in early or prehospital settings. In addition, in silico approaches can be used to strengthen discovery and development pipelines by enabling the targeting of toxin classes that have historically been difficult to neutralize. Importantly, these approaches are not mutually exclusive, but may be combined to achieve broader and more effective neutralization. At the same time, advances in toxicovenomics, structural biology, and diagnostic technologies are enabling increasingly detailed characterization of envenoming at the molecular level. This knowledge supports both the rational design of new therapies and their more precise clinical deployment. However, significant challenges remain, including achieving sufficient breadth of coverage, translating preclinical findings into clinical efficacy, and developing cost‐effective manufacturing and distribution strategies suitable for resource‐limited settings. Realizing the potential of new types of envenoming therapies will also require sustained investment, appropriate regulatory pathways, and coordinated partnerships among academia, industry, public health institutions, funders, and manufacturers to support clinical development, technology transfer, and equitable deployment in regions where snakebite envenoming is most prevalent. Overall, the field is progressing toward a more integrated and multidisciplinary framework, in which therapeutic design, diagnostics, and implementation are more closely aligned. While further scientific, regulatory, manufacturing, and implementation efforts are required to translate these advances into widely accessible treatments, new types of envenoming therapies have the potential to substantially improve outcomes and reduce the global burden of snakebite envenoming.

## Funding

A.H.L. is supported by a grant from Wellcome [221702/Z/20/Z].

## Conflicts of Interest

The author has declared that no conflicts of interest exist.
